# B12 deficiency or thrombotic microangiopathy? The key is in the peripheral smear

**DOI:** 10.1002/jha2.644

**Published:** 2023-01-27

**Authors:** Nelly Alanbari, Quentin Amiot, Julien Zuber, Idris Boudhabhay

**Affiliations:** ^1^ Department of Nephrology and Transplantation, Necker Enfants‐Malades University Hospital Assistance Publique des Hôpitaux de Paris Paris France; ^2^ Université Paris Cité Paris France; ^3^ Laboratory of Hematology, Necker Enfants‐Malades University Hospital Assistance Publique Des Hôpitaux de Paris Paris France

1

A 56‐year‐old patient, who had undergone a kidney transplant for systemic sarcoidosis 6 years ago, was admitted to our service for unexplained weight loss and asthenia. His immunosuppressive regimen was associated with low‐dose corticosteroid, tacrolimus, and mycophenolate mofetil. Complete blood count revealed a low platelet count of 110 × 10^9^/L and a hemoglobin level of 6 g/dl associated with an elevated mean corpuscular volume of 105 fl, but low reticulocyte counts of 36 × 10^9^/L. Thrombotic microangiopathy (TMA) was suspected because of increased lactate dehydrogenase levels (1250 IU/L) and undetectable haptoglobin associated with schistocytes (2%), suggestive of Intravascular hemolysis.

However, peripheral smear revealed macrocytosis, elliptocytes, and hypersegmented neutrophils (Figure 1), suggestive of B12 deficiency. Vitamin B12 levels were undetectable, confirming the diagnosis of pseudo‐TMA caused by B12 deficiency, as described in previous case reports [1, 2, 3]. Neurological examination was normal. Upper endoscopy showed gastric atrophy suggestive of pernicious anemia, and antral biopsy revealed gastric adenocarcinoma.

**FIGURE 1:**
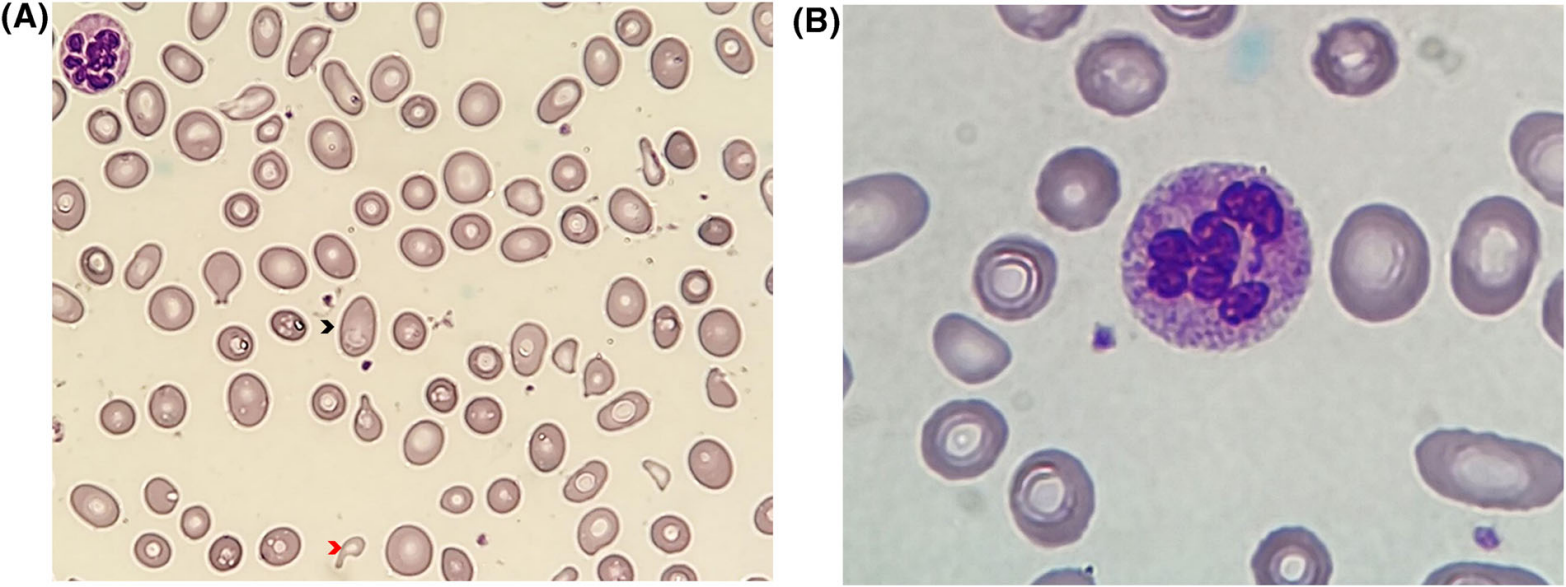
Light microscopy of peripheral blood smear in a patient with severe B12 deficiency. (A) Red blood cell fragments (red arrowhead) together with enlarged red blood cells with an oval shape (oval macrocytosis, black arrowhead) and (B) hypersegmented polymorphonuclear, suggestive of B12 deficiency

The patient was treated with intramuscular vitamin B12, 100 μg per day for 10 days, then every month. Reticulocyte response occurred 5 days later, and complete blood count normalized in 3 weeks.

In this case, an early diagnosis of pseudo‐TMA was made possible thanks to collaboration between biologists and clinicians with critical clinical impact, as it led to administration of vitamin B12 instead of unnecessary plasmapheresis.

## CONFLICT OF INTEREST

The authors declare no conflict of interest.

2

## ETHICAL AGREEMENT

The patient gave his written informed consent for publication.

## Data Availability

Data sharing is not applicable to this article as no new data were created or analyzed in this study.
